# Effects of Neighborhood Deprivation Index on Survival in Gastroesophageal Adenocarcinoma

**DOI:** 10.3390/healthcare13182296

**Published:** 2025-09-13

**Authors:** Sawyer Bawek, Mrinalini Ramesh, Malak Alharbi, Nour Nassour, Kayla Catalfamo, Han Yu, Beas Siromoni, Deepak Vadehra, Sarbajit Mukherjee

**Affiliations:** 1 Department of Internal Medicine, University at Buffalo, Buffalo, NY 14215, USA; sbawek@buffalo.edu (S.B.); mramesh3@buffalo.edu (M.R.); nournass@buffalo.edu (N.N.); 2 Department of Medical Oncology, Roswell Park Comprehensive Cancer Center, Buffalo, NY 14263, USA; malak.alharbi@roswellpark.org (M.A.); deepak.vadehra@roswellpark.org (D.V.); 3 Department of Internal Medicine, King Abdulaziz University, Jeddah 21589, Saudi Arabia; 4 Department of Biostatistics and Bioinformatics, Roswell Park Comprehensive Cancer Center, Buffalo, NY 14263, USA; kayla.catalfamo@roswellpark.org (K.C.); han.yu@roswellpark.org (H.Y.); 5 School of Health Sciences, University of South Dakota, Vermillion, SD 57069, USA; beassiromoni@gmail.com; 6 Department of Medical Oncology, Miami Cancer Institute, Baptist Health South Florida, Miami, FL 33176, USA

**Keywords:** gastric, esophageal, gastroesophageal, adenocarcinoma, neighborhood deprivation index

## Abstract

Previous studies linked disadvantaged neighborhoods to poor cancer outcomes. The Neighborhood Deprivation Index (NDI) quantifies socioeconomic disadvantage, but its impact on gastroesophageal adenocarcinoma outcomes remains understudied. We conducted a retrospective analysis of 40,589 patients with esophageal or gastric adenocarcinoma from the SEER database (1996–2015), stratifying them by NDI: less disadvantaged (NDI < 60) and highly disadvantaged (NDI ≥ 60). Multivariate regression showed NDI ≥ 60 was independently associated with worse overall survival (OS) (HR 1.027, *p* = 0.017) and disease-specific survival (DSS) (HR 1.025, *p* = 0.04). Other predictors of poor OS and DSS included older age (≥60 years old), male sex, single marital status, lack of insurance, advanced stage/grade, and gastric tumor site. In contrast, Hispanic and non-Hispanic Black ethnicity, urban residence, and undergoing surgery were associated with better outcomes. Disadvantaged neighborhoods are linked to poorer survival in upper GI cancers, likely due to socioeconomic barriers. Addressing social determinants of health is crucial to reducing these disparities.

## 1. Introduction

Gastroesophageal cancers account for 1.6 million new cases per year worldwide and claim nearly 1.3 million lives annually [1]. Social determinants of health (SDOH) play a crucial role in cancer survival, especially gastric cancer, as they directly influence access to timely diagnosis, treatment, and care. Socioeconomic status (SES), education, occupation, geographic location, and living conditions shape healthcare access and outcomes [2,3,4].

The Neighborhood Deprivation Index (NDI) has been used to quantify SES. Several studies have highlighted the correlation between disadvantaged living conditions and worse outcomes in various cancers [5,6,7,8,9]. Low-income individuals often face barriers to early screening, treatment, and follow-up care, which can lead to later-stage diagnoses and reduced survival [10].

Racial and ethnic backgrounds further complicate survival disparities in gastric cancer. Research shows that minority groups such as Hispanic, Asian, Pacific Islander, and Black populations have higher rates of gastric cancer compared to non-Hispanic White individuals [11]. Recent investigations have also shown that patients of Asian and Pacific Islander descent were more likely to present with earlier-stage disease and have better survival than White patients [12]. Whereas Black and Hispanic patients are more likely to present with advanced-stage disease and have worse survival outcomes compared to non-Hispanic White patients, with contributing factors including differences in tumor biology, access to care, insurance status, and social determinants of health [13]. Moreover, Asian gastric cancer patients have been found to have a 12% higher survival rate, primarily attributed to earlier diagnosis. The survival advantage observed in Asian patients may partially be explained by differences in tumor subtype, response to therapy, and extent of surgical treatment, but these factors do not fully account for the observed disparities, indicating a complex interplay of biological, environmental, and healthcare system factors [14,15].

Only a limited number of epidemiological studies have investigated the association of SES and geographical variation in relation to survival outcomes for patients with gastroesophageal adenocarcinoma. Understanding the impact of SES conditions and outcomes is important to developing strategies to mitigate healthcare disparities in this patient population. In this study, we aimed to investigate the effect of NDI among patients with gastroesophageal adenocarcinoma.

## 2. Materials and Methods

In this retrospective analysis, we included patients diagnosed with gastroesophageal adenocarcinoma (esophageal, gastric, and gastroesophageal junction) from 1996 to 2015. We extracted demographics, marital status, insurance, clinical characteristics including tumor histology, grade, stage, surgical history, and survival outcomes data using the Surveillance, Epidemiology, and End Results (SEER) database [16]. We excluded patients with missing survival follow-up data. NDI was divided into two categories: NDI < 60%, representing the least disadvantaged areas, and NDI ≥ 60% representing the most disadvantaged areas. The NDI cutoff at 60 was derived from a study by Roy et al. [4]. Demographic and clinical characteristics were summarized by NDI using frequencies and relative frequencies for categorical variables and median and interquartile range for continuous variables. All associations were compared using Kruskal–Wallis and chi-square tests.

Finally, survival outcomes, including time-to-event outcomes, overall survival (OS), and disease-specific survival (DSS), were summarized by NDI using Kaplan–Meier curves. All associations were compared using the log-rank test. Median values and 95% confidence intervals were reported. We constructed a multivariate Cox model to evaluate the effect of NDI on the OS/DSS concurrently with the other variables like age, sex, race, location, marital status, insurance, disease grade, surgery, and year at diagnosis (one variable at a time). To further assess the treatment effect over time, we also applied the Restricted Mean Survival Time (RMST) regression, which provides an alternative measure of survival by calculating the area under the survival curve up to 120 months. A linear link function was utilized. The analysis was conducted in R v4.2.3 at a significance level of ≤0.05. The RMST regression was conducted using PROCRMSTREG, SAS 9.4.

## 3. Results

A total of 40,589 patients diagnosed with gastroesophageal adenocarcinoma were analyzed, comprising 41.6% (n = 16,898) with NDI ≥ 60 and 58.4% (n = 23,691) with NDI < 60. Overall, 21,384 patients had esophageal and 19,205 had gastric adenocarcinoma. Patients from disadvantaged areas (NDI ≥ 60) were more often Black or Hispanic, single, uninsured, lived in rural areas, presented with more advanced disease (stage III/IV), and were less likely to undergo surgery compared with NDI < 60 (*p* < 0.001) (Table 1).

**Figure 1:**
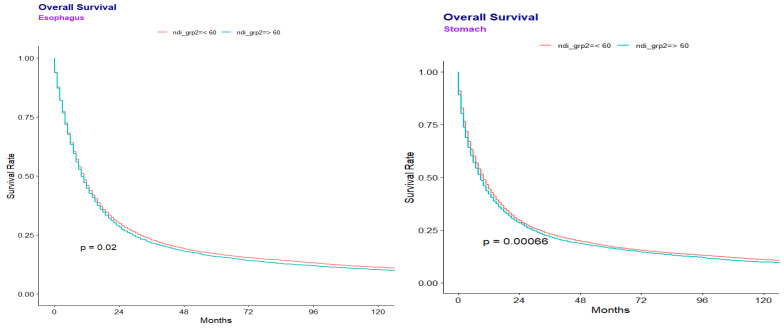
Esophageal and gastric overall survival and disease specific survival.

Median overall survival (OS) was lower in disadvantaged areas for gastric cancer: 9.0 months (NDI ≥ 60) vs. 10.0 months (NDI < 60) (*p* = 0.0007). Similar results were seen for esophageal cancer at 10.0 months (NDI ≥ 60) vs. 11.0 months (NDI < 60) (*p* = 0.020). (Figure 1). Multivariate regression analysis for the combined gastroesophageal adenocarcinoma cohort showed that NDI ≥ 60 was independently associated with worse OS (HR 1.027, *p* = 0.017) (Table 2).

Median DSS was also lower in more disadvantaged areas. Patients with gastric cancer from more disadvantaged areas had a shorter DSS at 10.0 months compared to 12.0 months for those living in less disadvantaged areas (*p* = 0.010). Patients with esophageal cancer had a similar median DSS at 12.0 months, regardless of what area they lived in. Multivariate analysis showed that NDI ≥ 60 was independently associated with DSS (HR 1.025, *p* = 0.04).

Other significant predictors of worse OS and DSS included age (60+), male sex, single marital status, being uninsured, advanced disease stage (III/IV), advanced disease grade (III/IV), and gastric disease site (*p* < 0.0001). Hispanic patients, patients who lived in an urban area, and patients who had surgery had improved OS and DSS (*p* < 0.0001) (Table 2). RMST analysis further confirmed these findings.

## 4. Discussion

Our study highlights the significant impact of socioeconomic factors on survival outcomes in individuals with gastroesophageal adenocarcinoma. It also demonstrates that patients with gastroesophageal adenocarcinoma who live in disadvantaged areas (NDI ≥ 60) had significantly worse OS and DSS compared to individuals living in less disadvantaged areas, even after adjusting for other variables like demographic and clinical factors.

Our results are consistent with prior studies [5,6,7,8,9]. Similar studies have shown that higher socioeconomic deprivation is associated with lower odds of receiving chemotherapy and worse OS in patients with metastatic gastric cancer [8]. The study showed that patients with the highest deprivation quartile had a median OS of 5.1 months compared to 7.5 months in the lowest deprivation quartile [8]. Another study found that there was an increased 30-day mortality following esophagectomy in patients with higher deprivation when compared to the lowest deprivation quintiles, with an odds ratio of 1.37 [17]. These studies highlight the impact of social determinants of health on survival outcomes.

Multiple factors likely contribute to these disparities. Examples include delays in diagnosis and treatment, lower health literacy, and worse access to healthcare. Our study showed that disadvantaged areas had a higher proportion of uninsured patients from rural areas with limited access to specialized care and were more likely to present with an advanced disease stage. Our project did show a significantly higher proportion of patients that were insured in the high-NDI group in comparison to the low. Similar investigations into the impact of neighborhood deprivation on survival have shown comparable results [18]. This is potentially attributed to the increased number of Medicaid patients in areas with the highest neighborhood disadvantage, as noted by Goel et al. [18]. Tailored healthcare policies that aim to improve healthcare delivery to areas with high deprivation may help improve outcomes for patients with gastroesophageal adenocarcinoma. For instance, Kronfli et al. found that targeted interventions focused on pain management, transportation, and financial aid at an inner-city academic center, particularly among Black patients, could improve cancer outcomes and quality of life [17]. Similarly, Lineback et al. found that social workers and medical advocates who work individually with patients can improve care for patients with esophageal cancers. Specifically, individuals with low SES were more likely to have greater difficulties with caregiver communication, trust, and financial strain. The study also showed individuals with lower SES were less likely to understand their treatment options well. When provided with a care team manager or social worker, hospital systems were able to help solve these issues. These professionals can help navigate complex treatments and financial concerns and help improve clinical outcomes [19]. As well, patients from high-NDI groups have been shown to have reduced access to high-quality healthcare, often undergoing emergency surgeries and having poor primary care [20,21]. These disparities in access to treatment options could contribute to the difference in survival outcomes in low- versus high-NDI groups. Future directions would include studying the extent to which low versus high NDI groups have access to advancements in treatment and their impact on survival.

This is the first study to analyze the impact of NDI on gastroesophageal adenocarcinoma. One strength of our study is utilizing a large, nationally representative SEER database, which covers more than 50% of the US population. Our findings demonstrate a gradual improvement in both OS and DSS of gastroesophageal adenocarcinoma over time, with progressively reduced hazard ratios for patients diagnosed in 1990–2000, 2001–2010, and 2011+ compared with those diagnosed in 1975–1989 (Table 2).

Our study has several limitations. Firstly, the SEER database does not have details on individual socioeconomic factors such as income, education, zip codes, subcategories of insurance, and employment status, which limits our complete understanding of the disparities among the patient population. Additionally, details on environmental influences like air quality, water pollution, transportation, and treatment regimens are unavailable. Future prospective cohort studies are needed to further understand these disparities.

## 5. Conclusions

In conclusion, we showed that patients from disadvantaged areas (NDI ≥ 60) have significantly worse OS and DSS for gastroesophageal adenocarcinoma, even after adjusting for demographic and clinical variables. Patients’ survival is due to the complex interplay of social determinants of health and racial/ethnic background. Disparities in survival outcomes reflect systemic factors, such as access to healthcare, and individual factors, such as stage at diagnosis and response to treatment. Understanding these factors is essential for developing strategies to mitigate healthcare disparities and improve outcomes for diverse populations. Addressing socioeconomic and cultural barriers, as well as improving access to early detection and treatment, is crucial for reducing the survival gap among gastroesophageal adenocarcinoma patients.

## Figures and Tables

**Table 1:** Overall sample demographics separated by NDI.

Overall Sample		<60	≥60	Overall	*p*-Value
		23,691	16,898	40,589	
Age	Mean	66.17	65.9	66.06	0.01003
	Median (IQR)	67.0 (58.0, 75.0)	66.0 (57.0, 75.0)	66.0 (58.0, 75.0)	
Age	<40	513 (2.165%)	357 (2.113%)	870 (2.143%)	0.1404
	40–50	1779 (7.509%)	1242 (7.350%)	3021 (7.443%)	
	50–60	4710 (19.88%)	3516 (20.81%)	8226 (20.27%)	
	60+	16,689 (70.44%)	11,783 (69.73%)	28,472 (70.15%)	
Sex	Female	5229 (22.07%)	3679 (21.77%)	8908 (21.95%)	0.4688
	Male	18,462 (77.93%)	13,219 (78.23%)	31,681 (78.05%)	<0.001
Race	Hispanic	1776 (7.497%)	2234 (13.22%)	4010 (9.880%)	
	Non-Hispanic Black	1131 (4.774%)	1521 (9.001%)	2652 (6.534%)	
	Non-Hispanic White	19,738 (83.31%)	12,543 (74.23%)	32,281 (79.53%)	
	Other	1046 (4.415%)	600 (3.551%)	1646 (4.055%)	
Location	Rural	4673 (19.72%)	4982 (29.48%)	9655 (23.79%)	<0.001
	Urban	19,018 (80.28%)	11,916 (70.52%)	30,934 (76.21%)	
Marital Status	Single	8642 (36.48%)	6776 (40.10%)	15,418 (37.99%)	<0.001
	Married	15,049 (63.52%)	10,122 (59.90%)	25,171 (62.01%)	
Insurance Status	Insured	8659 (36.55%)	7523 (44.52%)	16,182 (39.87%)	<0.001
	Uninsured	339 (1.431%)	345 (2.042%)	684 (1.685%)	
	Unknown	14,693 (62.02%)	9030 (53.44%)	23,723 (58.45%)	
Disease Stage	I/II	4560 (19.25%)	3931 (23.26%)	8491 (20.92%)	<0.001
	III/IV	7065 (29.82%)	5917 (35.02%)	12,982 (31.98%)	
	Unknown	12,066 (50.93%)	7050 (41.72%)	19,116 (47.10%)	
Grade	I/II	8460 (35.71%)	6331 (37.47%)	14,791 (36.44%)	<0.001
	III/IV	11,767 (49.67%)	7980 (47.22%)	19,747 (48.65%)	
	Unknown	3464 (14.62%)	2587 (15.31%)	6051 (14.91%)	
Surgery	No	10,604 (44.76%)	8887 (52.59%)	19,491 (48.02%)	<0.001
	Yes	11,768 (49.67%)	7368 (43.60%)	19,136 (47.15%)	
	Unknown	1319 (5.568%)	643 (3.805%)	1962 (4.834%)	
Disease Site	Esophagus	12,554 (52.99%)	8830 (52.25%)	21,384 (52.68%)	0.1504
	Stomach	11,137 (47.01%)	8068 (47.75%)	19,205 (47.32%)	

**Table 2:** Multivariate regression analysis for OS and DSS.

Overall Sample (OS)		HR	*p*-Value	Overall Sample (DSS)	HR	*p*-Value
NDI	≥60	1.04	0.017		1.04	0.041
Age	60+	1.45	<0.0001		1.24	<0.0001
Sex	Male	1.06	<0.0001		1.03	0.023
Race	Non-Hispanic Black	1.01	<0.0001		0.99	<0.0001
	Hispanic	0.96	<0.0001		0.96	<0.0001
Location	Urban	0.94	<0.0001		0.93	<0.0001
Marital Status	Single	1.20	<0.0001		1.16	<0.0001
Insurance	Uninsured	1.22	<0.0001		1.20	<0.0001
Disease Stage	III/IV	2.02	<0.0001		2.30	<0.0001
Disease Grade	III/IV	1.33	<0.0001		1.40	<0.0001
Disease Site	Stomach	1.21	<0.0001		1.20	<0.0001
Surgery	Yes	0.37	<0.0001		0.34	<0.0001
Year at Diagnosis	1990–2000 2001–2010 2011+	0.87 0.72 0.64	<0.0001		0.85 0.69 0.61	<0.0001

## Data Availability

All the data used for this study is from the publicly available Surveillance, Epidemiology, and End Results (SEER) database at https://seer.cancer.gov/data/index.html (accessed on 1 February 2025).
